# Supplementary material for: Asymmetric cell division shapes naive and virtual memory T-cell immunity during ageing
Source: Nat Commun. 2021 May 11;12:2715. doi: 10.1038/s41467-021-22954-y (PMC8113513; doi:10.1038/s41467-021-22954-y)
Supplement: Supplementary file 1 — Supplementary Information [file 41467_2021_22954_MOESM1_ESM.pdf]

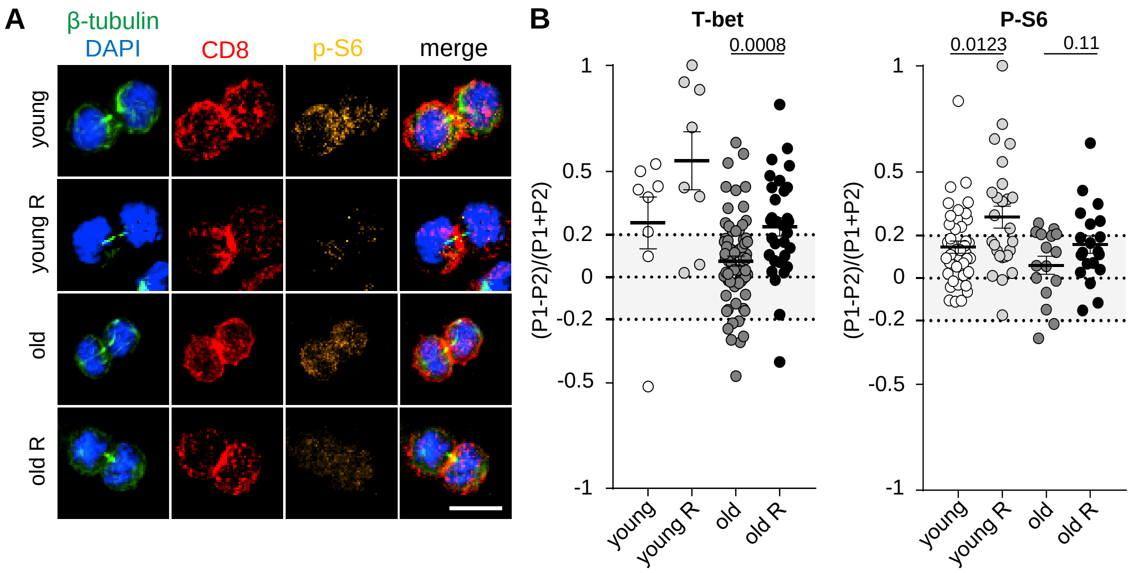

Supplementary Figure 1. Transient mTOR inhibition leads to higher asymmetric cell division rates as measured by the polarization of T-bet and phospho-S6. Transient mTOR inhibition was achieved by treatment with rapamycin (20nM). Cells were exposed to drug treatment from 12 hours post-activation until fixation for confocal microscopy analysis. Stimulation was done on human Fc-ICAM, α-CD3 and α-CD28 coated plates for 32-36 hours. (A) Confocal microscopy images from naïve CD8$^+$ T cells obtained from young or aged P14 mice. Scale bar=10μm. (B) Polarization of T-bet (young, n=8; young R, n=8; old, n=71; old R, n=31), and P-S6 (young, n=41; young R, n=26; old, n=16; old R, n=20) is higher upon transient rapamycin treatment for CD8$^+$ T cells isolated from both young and old P14 mice. Data are represented as mean ± SEM. Pooled data from 2 experiments. Age of the animals: experiment 1 (40-43 weeks), experiment 2 (71 weeks). Statistical analysis was performed using the unpaired two-tailed Student`s *t* test. Exact *P* values are depicted in the figure.

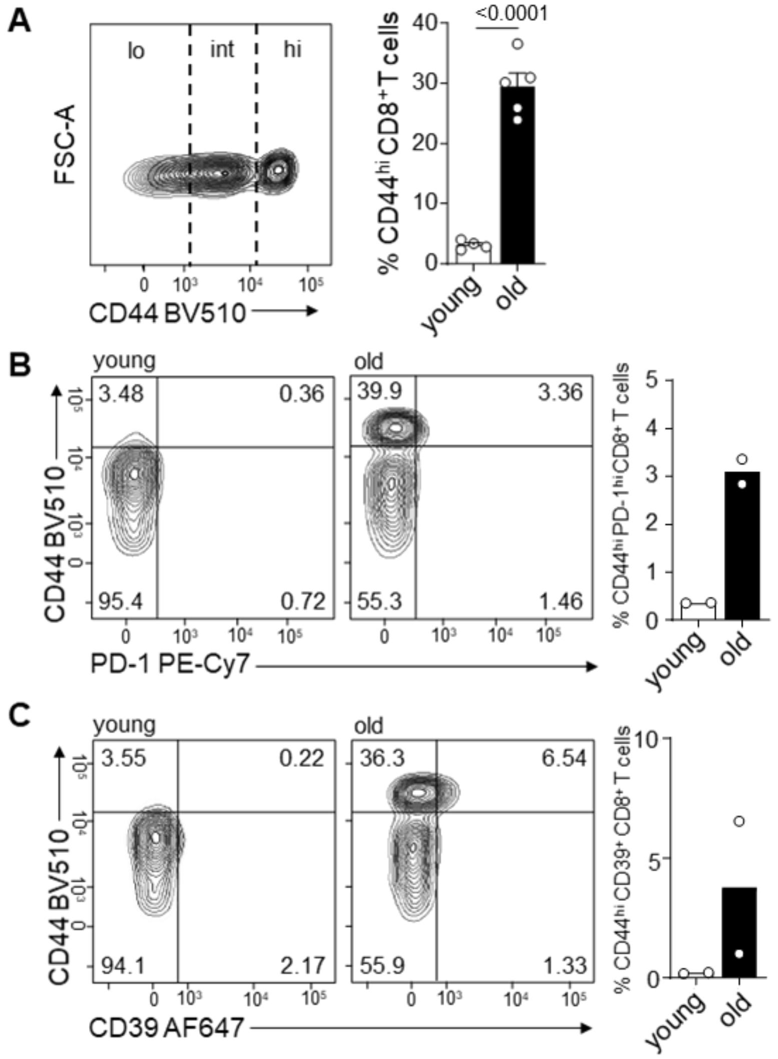

Supplementary Figure 2. CD44^{hi} CD8^{+} T cells observed in higher frequencies in aged mice show a modest increase in exhaustion. (A) Gating strategy used to identify CD44^{lo}, CD44^{int} and CD44^{hi} cells. CD44^{hi} CD8^{+} T cells are present in higher frequencies in the blood of aged P14 mice (70, 90 or 100 weeks-old). Pooled data from 2 independent experiments (young, n=4; old, n=5). (B) Representative flow cytometry plots of CD44 and PD-1 expressing cells in the blood of young and aged P14 mice (95 or 102 weeks-old) (left panel). Frequencies of PD-1^{hi} CD44^{hi} CD8^{+} T cells in the blood of young and old P14 mice (right panel). (C) Representative plots of CD44 and CD39 expressing cells in the blood of young and old P14 mice (left panel). Frequencies of CD44^{hi} CD8^{+} T cells expressing CD39 in the blood of young and old P14 mice (right panel). (B-C) Representative data from 1 experiment (young, n=2; old, n=2). Data are depicted as mean + SEM. Statistical analysis was performed using the unpaired two-tailed Student's *t* test. Exact *P* values are depicted in the figure.

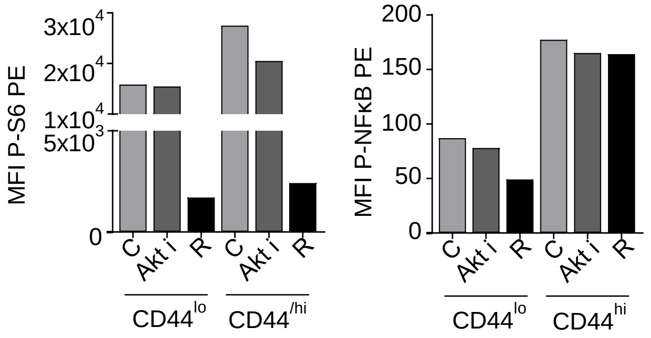

Supplementary Figure 3. Phosphorylation of downstream targets of mTORC1 (S6) and mTORC2 (NFκB). Naïve and TVM cells from young and aged (70 weeks-old) P14 mice were stimulated *in vitro* on human Fc-ICAM-1, α-CD3, α-CD28 coated plates with or without transient mTOR inhibition. 36 hours post-stimulation, cells were assessed for their P-S6 and P-NFκB expression levels by flow cytometry.

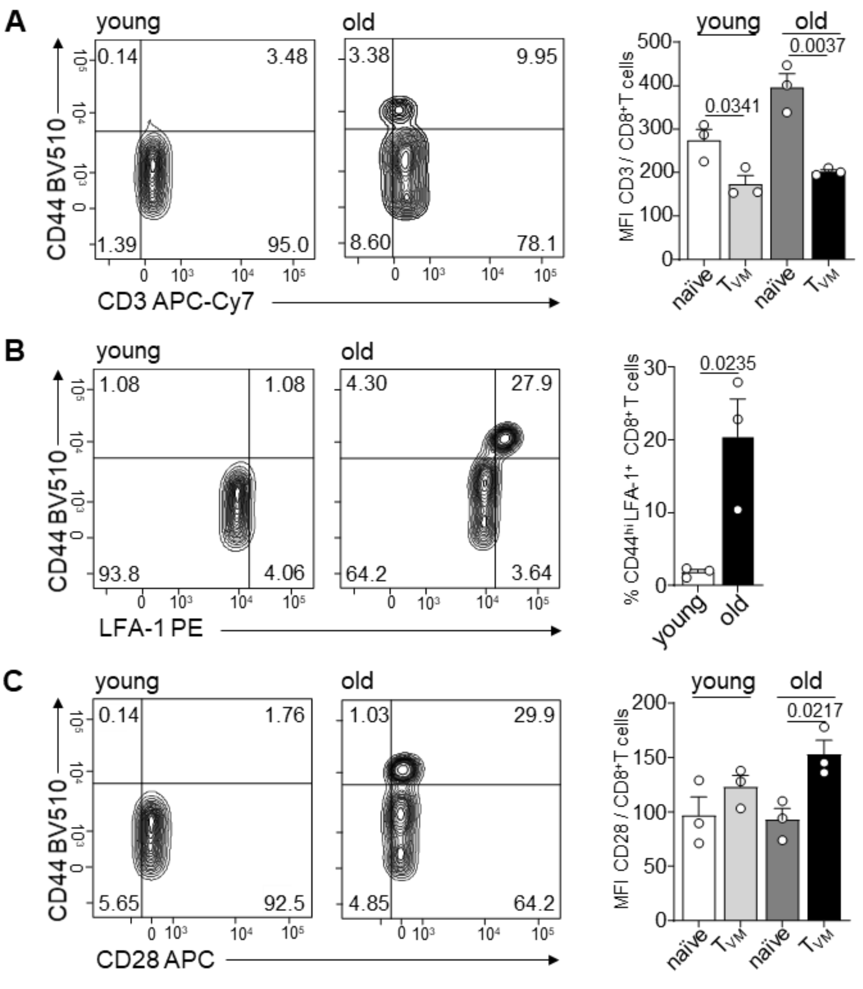

Supplementary Figure 4. Altered expression of immune synapse components in T_{VM} CD8⁺ from old P14 mice. (A) Representative plots of CD44 and CD3 expression on $CD8^+$ T cells in the blood of young and old P14 mice (74, 87 or 91 weeks-old) (left panel), MFI of CD3 expression in $CD44^{lo}$ and $CD44^{hi}$ $CD8^+$ T cells (right panel). (B) Representative plots of CD44 and LFA-1 expression on $CD8^+$ T cells in the blood of young and old P14 mice (left panel). Frequencies of LFA-1 expressing cells is higher in $CD44^{hi}$ $CD8^+$ T cells (right panel). (C) Representative plots of CD44 and CD28 in the blood of young and old P14 mice (left panel). MFI of CD28 expression in $CD44^{lo}$ and CD44hi $CD8^+$ T cells (right panel). Data obtained from 1 experiment (n=3), and depicted as mean + SEM. Statistical analysis was performed using the unpaired two-tailed Student's *t* test. Exact *P* values are depicted in the figure.

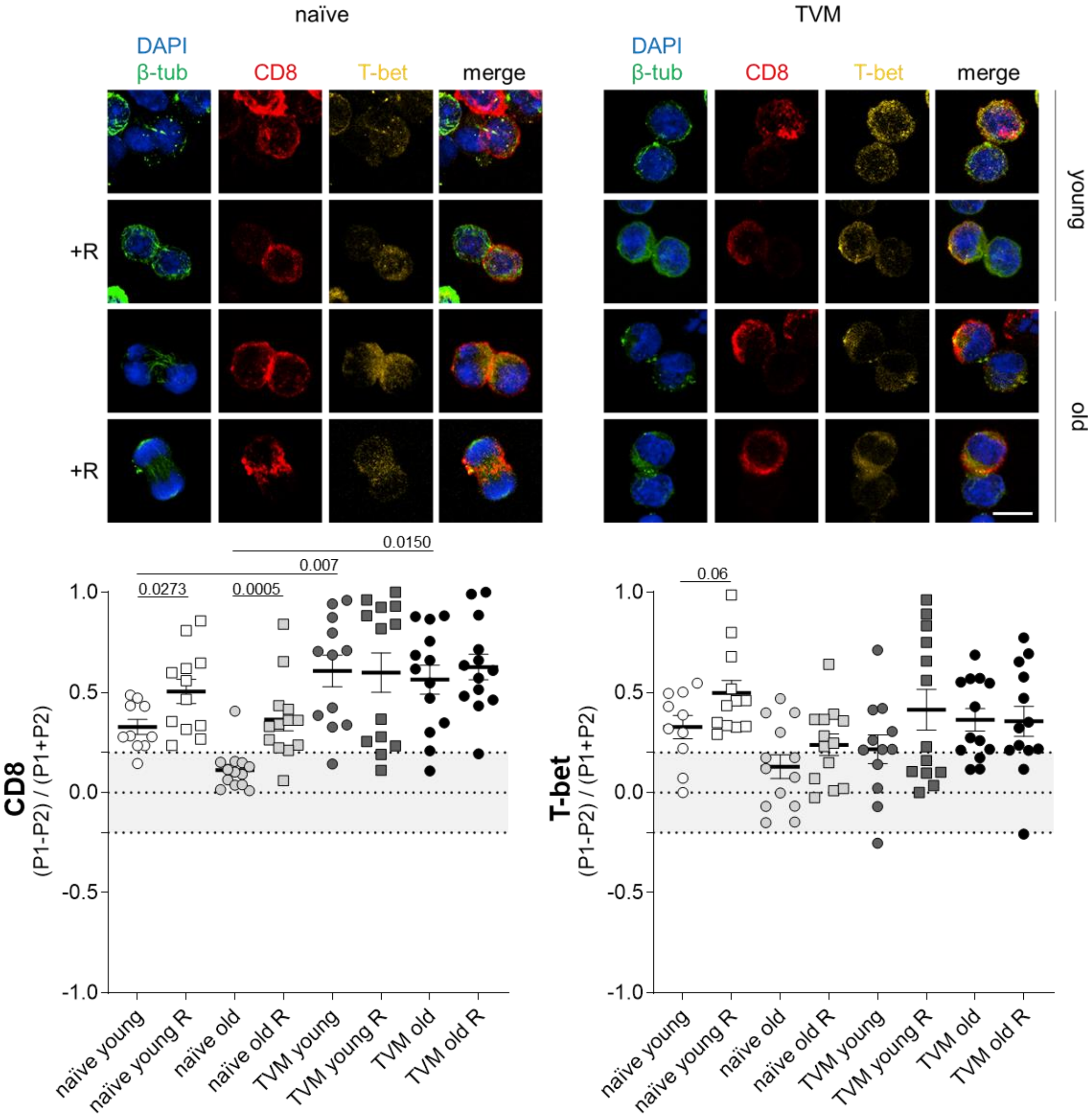

Supplementary Figure 5. **T_{VM} CD8⁺ T cells from polyclonal wild type C57BL/6 mice show intrinsically high ACD rates.**

Upper panel: Confocal images from murine naïve and T_{VM} wt B6 CD8⁺ T cells fixed 36-40h after *in vitro* stimulation on α-CD3, α-CD28 and human Fc-ICAM-1 coated wells in presence or not of transient mTOR inhibition by rapamycin (R) (20nM) (naïve young, n=10; naïve young R, n=12; naïve old, n=13; naïve old R, n=12; TVM young, n=12; TVM young R, n=13; TVM old, n=13; TVM old R, n=13). Scale bar=10µm. Cells were exposed to rapamycin from 12h post-activation until fixation for confocal microscopy analysis. Lower panel: CD8 and T-bet asymmetry rates in naïve and T_{VM} CD8⁺ T cells isolated from young or old wt B6 mice. Data are shown as mean ± SEM. Data obtained from 1 experiment. Aged mice: 100 or 104 weeks-old. For each experimental group (young or old) sorted cells from at least 3 animals were pooled. Statistical analysis was performed using the unpaired two-tailed Student's t test. Exact *P* values are depicted in the figure.

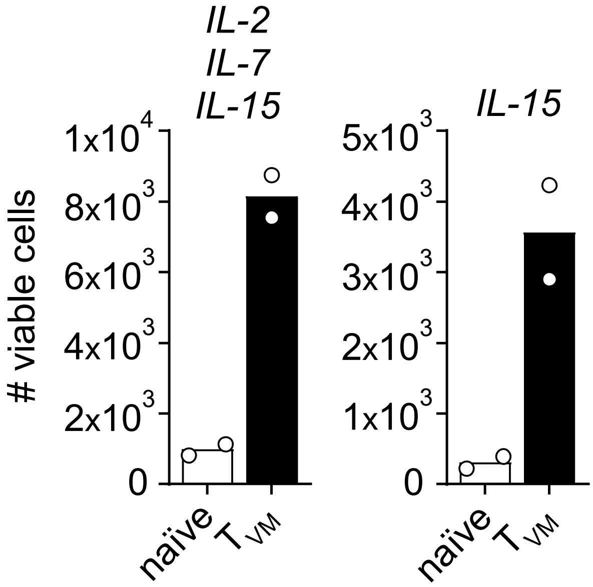

Supplementary Figure 6. T$_{VM}$ CD8$^+$ T cells from aged mice are better survivors under limiting concentrations of cytokines. Isolated naïve or T$_{VM}$ CD8$^+$ T cells from aged mice (>70 weeks) were cultured in T cell medium supplemented with human IL-2, and stimulated on α-CD3 (5 µg/ml) (BioLegend), α-CD28 (5 µg/ml) (BioLegend), and human Fc-ICAM-1 (50 µg/ml) (R&D Biosciences) for 30-36 hours. After plate-bound stimulation, cells were harvested, washed in PBS, and transferred to uncoated new wells containing limited concentrations of recombinant mouse IL-15 or a cocktail containing IL-2, IL-7, IL-15 (<1 ng/ml). Cells were analyzed for viability by flow cytometry 7 days later. Data representative of 1 out of 2 experiments with technical duplicates.

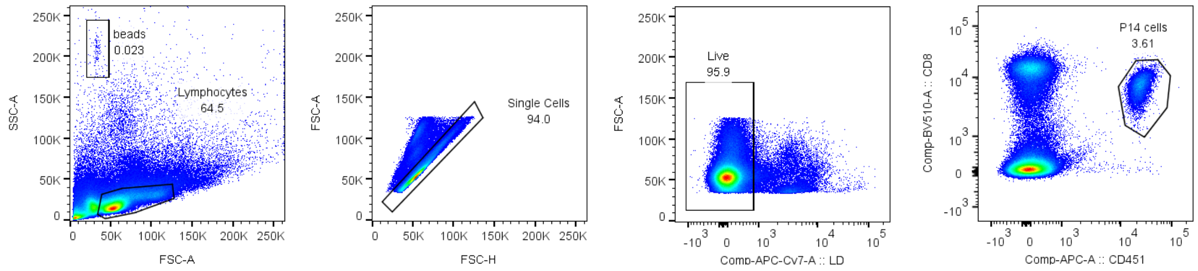

Supplementary Figure 7: Flow cytometry gating strategy.

For all samples the following gating strategy was used: lymphocytes (SSC-A/FSC-A), exclusion of doublets (FSC-A/FSC-H), live cells (FSC-A/Live/Dead marker Near-IR). To identify congenitally marked P14 TCR transgenic CD8$^+$ T cells, cells were gated for CD8 and for the congenic marker CD45.1.
