# Supplementary material for: Asymmetric cell division shapes naive and virtual memory T-cell immunity during ageing
Source: Nat Commun. 2021 May 11;12:2715. doi: 10.1038/s41467-021-22954-y (PMC8113513; doi:10.1038/s41467-021-22954-y)
Supplement: Supplementary file 3 — Reporting Summary [file 41467_2021_22954_MOESM3_ESM.pdf]

nature research

Corresponding author(s): Annette Oxenius

Last updated by author(s): 28/02/2021

Reporting Summary

Nature Research wishes to improve the reproducibility of the work that we publish. This form provides structure for consistency and transparency in reporting. For further information on Nature Research policies, see our Editorial Policies and the Editorial Policy Checklist.

Statistics

For all statistical analyses, confirm that the following items are present in the figure legend, table legend, main text, or Methods section.

n/a	Confirmed	
☐	☒	The exact sample size (*n*) for each experimental group/condition, given as a discrete number and unit of measurement
☐	☒	A statement on whether measurements were taken from distinct samples or whether the same sample was measured repeatedly
☐	☒	The statistical test(s) used AND whether they are one- or two-sided<br>*Only common tests should be described solely by name; describe more complex techniques in the Methods section.*
☒	☐	A description of all covariates tested
☐	☒	A description of any assumptions or corrections, such as tests of normality and adjustment for multiple comparisons
☐	☒	A full description of the statistical parameters including central tendency (e.g. means) or other basic estimates (e.g. regression coefficient) AND variation (e.g. standard deviation) or associated estimates of uncertainty (e.g. confidence intervals)
☐	☒	For null hypothesis testing, the test statistic (e.g. *F*, *t*, *r*) with confidence intervals, effect sizes, degrees of freedom and *P* value noted<br>*Give P values as exact values whenever suitable.*
☒	☐	For Bayesian analysis, information on the choice of priors and Markov chain Monte Carlo settings
☒	☐	For hierarchical and complex designs, identification of the appropriate level for tests and full reporting of outcomes
☒	☐	Estimates of effect sizes (e.g. Cohen's *d*, Pearson's *r*), indicating how they were calculated

Our web collection on statistics for biologists contains articles on many of the points above.

Software and code

Policy information about availability of computer code

Data collection	Data were collected using BD FACSDiva (v8.0.1), a Visitron Confocal System or a Nikon A1R confocal system.
Data analysis	Flow cytometry data were analyzed by Flowjo 10.7.1. Microscopy analysis was performed using Volocity software (version 6.3.0, PerkinElmer or Volocity® Quantitation, QuorumTechnologies). Data was exported into Graphpad prism (version 7.0 or 8.2.0) for statistical analysis.

For manuscripts utilizing custom algorithms or software that are central to the research but not yet described in published literature, software must be made available to editors and reviewers. We strongly encourage code deposition in a community repository (e.g. GitHub). See the Nature Research guidelines for submitting code & software for further information.

Data

Policy information about availability of data

All manuscripts must include a data availability statement. This statement should provide the following information, where applicable:

- Accession codes, unique identifiers, or web links for publicly available datasets
- A list of figures that have associated raw data
- A description of any restrictions on data availability

Additional flow cytometry and imaging data supporting the findings are available from the first/corresponding author upon request. Source data for Figures 1B; 2B-G; 3A, C; 4C-D; 5B-D and Supplementary Figures S1B, S2, S4, S5 and S6 are available in the Source Data File.

Field-specific reporting

Please select the one below that is the best fit for your research. If you are not sure, read the appropriate sections before making your selection.

- [x] Life sciences
- [ ] Behavioural & social sciences
- [ ] Ecological, evolutionary & environmental sciences

For a reference copy of the document with all sections, see nature.com/documents/nr-reporting-summary-flat.pdf

Life sciences study design

All studies must disclose on these points even when the disclosure is negative.

Sample size	For microscopy analysis, the totality of mitotic cells found in each sample were imaged/acquired. The numbers varied depending on the abundance of the subsets analyzed, their proliferation potential, and the quality of the preparation prior to imaging. We made bigger efforts to increase sample size when performing novel analysis: comparison of asymmetric cell division rates between CD8 T cells from young vs. aged mice (Fig. 1), or when these cells were further dissected in subsets (naive and TVM) (Fig. 4). For these n>19. For functional readouts, 3-5 recipient mice were used per group within each experiment. This number was based on previous experience with the adoptive transfer and infection model (Borsa et al., Science Immunology 2019), the availability of the mice, and feasibility of the experiment. No statistical test was used to predetermine sample size.
Data exclusions	As mitotic cells were imaged based on the presence of condensed chromosomes and a tubulin bridge between two daughter-cells, we later excluded the images where the CD8 staining was not visible or very weak, which makes quantification unreliable. One mouse from the experiment represented in Figure 5 had to be excluded because the counting beads were not detected, which prohibited us to calculate cell numbers.
Replication	Concerning experiments represented in the main figures: (1) microscopy experiments were repeated at least 3 times, and (2) functional experiments were repeated at least twice with identical/very similar conditions. Part of the experiments in the supplementary material were done just once. The information is clearly found in the Figure legends. All attempts of replication were successful.
Randomization	Recipient mice were randomly distributed amongst groups at the start of each experiment.
Blinding	Blinding was not performed. For in vivo experiments it is required by local authorities to state on the cage cards all handling that is done to the mice. Investigators were not blinded during data collection and analysis.

Reporting for specific materials, systems and methods

We require information from authors about some types of materials, experimental systems and methods used in many studies. Here, indicate whether each material, system or method listed is relevant to your study. If you are not sure if a list item applies to your research, read the appropriate section before selecting a response.

Materials & experimental systems

n/a	Involved in the study	
[ ]	[x]	Antibodies
[ ]	[x]	Eukaryotic cell lines
[x]	[ ]	Palaeontology and archaeology
[ ]	[x]	Animals and other organisms
[x]	[ ]	Human research participants
[x]	[ ]	Clinical data
[x]	[ ]	Dual use research of concern

Methods

n/a	Involved in the study	
[x]	[ ]	ChIP-seq
[ ]	[x]	Flow cytometry
[x]	[ ]	MRI-based neuroimaging

Antibodies

Antibodies used

Fluorophore-conjugated antibodies used for flow cytometry staining were purchased from:

1. BD Biosciences
α-CD44 FITC IM7 (1/200); cat. number 553133; validation: Staining on C57BL/6 mouse splenocytes
α-IL-2 APC JES6-5H4 (1/100); cat. number 554429; validation: Staining on BALB/C PMA + Ionomycin stimulated (15h) splenocytes treated with brefeldin A and monensin
streptavidin APC (1/500); cat. number 554067; validation: tested during reagent development
streptavidin PE-Cy7 (1/500); cat. number 557598; validation: tested during reagent development

2. eBiosciences
α-IFNγ PE XMG1.2 (1/100); cat. number 12-7311-82; validation: Intracellular staining on PMA + Ionomycin stimulated (5h) C57BL/6 splenocytes treated with brefeldin A and monensin

α-KLRG1 PE-Cy7 2F1 (1/200); cat. number 25-5893-82; validation: Staining on C57BL/6 mouse splenocytes
α-PD-1 FITC J43 (1/100); cat. number 11-9985; validation: Staining on d3 stimulated (anti-CD3 + anti-CD28 antibodies) C57BL/6 mouse splenocytes

3. Cell Signalling
α-phospho-Akt PE D9E (1/50); cat. number 5315S; validation: Jurkat cells treated or not with PI3 Kinase inhibitors
α-phospho-NFκB PE 93H1 (1/50); cat. number 5733S; validation: HeLa cells treated or not with hTNF-α or Calyculin A
α-phospho-S6 PE D57.2.2E (1/50); cat. number 5316S; validation: Jurkat cells treated or not with PI3 Kinase inhibitors

4. BioLegend
α-B220 biotin RA3-6B2 (1/200); cat. number 103204; validation: Staining on C57BL/6 mouse splenocytes
α-CD28 APC 37.51 (1/200); cat. number 102109; validation: Staining on C57BL/6 mouse splenocytes
α-CD122 FITC TM-β1 (1/100); cat. number 123208; validation: Staining on C57BL/6 mouse splenocytes
α-CD3ε APC-Cy7 145-2C11 (1/100); cat. number 100330; validation: Staining on C57BL/6 mouse splenocytes
α-CD39 AF647 Duha59 (1/100); cat. number 143807: validation: Staining on C57BL/6 mouse splenocytes
α-CD44 BV510 IM7 (1/200); cat. number 103043; validation: Staining on C57BL/6 mouse splenocytes
α-CD4 biotin GK1.5 (1/200); cat. number 553648; validation: Staining on C57BL/6 mouse splenocytes
α-CD44 PE IM7 (1/200); cat. number 103008; validation: Staining on C57BL/6 mouse splenocytes
α-CD45.1 APC A20 (1/200); cat. number 110720; validation: Staining on C57BL/6 mouse splenocytes
α-CD45.1 PerCP A20 (1/200); cat. number 110726; validation: Staining on C57BL/6 mouse splenocytes
α-CD8 APC-Cy7 53-6.7 (1/100); cat. number 100714; validation: Staining on C57BL/6 mouse splenocytes
α-CD8 BV510 53-6.7 (1/200); cat. number 100752; validation: Staining on C57BL/6 mouse splenocytes
α-CD8 PerCP 53-6.7 (1/200); cat. number 100794; validation: Staining on C57BL/6 mouse splenocytes
α-CD49d biotin R1-2 (1/200); cat. number 103603; validation: Staining on C57BL/6 mouse splenocytes (+streptavidin)
α-CD62L PerCP MEL-14 (1/100); cat. number 104430; validation: Staining on C57BL/6 mouse bone marrow
α-CXCR3 BV421 CXCR3-173 (1/100); cat. number 126521; validation: Staining on C57BL/6 mouse splenocytes
α-KLRG1 FITC 2F1 (1/200); cat. number 138410; validation: Staining on C57BL/6 mouse splenocytes
α-IL-7Rα BV421 A7R34 (1/100); cat. number 135023; validation: Staining on C57BL/6 mouse splenocytes
α-LFA-1 PE 2D7 (1/200); cat. number 101107; validation: Staining on C57BL/6 mouse splenocytes
α-NKG2D APC CX5 (1/100); cat. number 130211; validation: Staining on C57BL/6 mouse splenocytes
α-PD-1 PE-Cy7 29F.1A12 (1/100); cat. number 135215; validation: Con-A stimulated C57BL/6 mouse splenocytes
α-TNF FITC MP6-XT22 (1/100); cat. number 506304; validation: Intracellular staining on PMA + Ionomycin stimulated (5h) C57BL/6 splenocytes treated with brefeldin A and monensin
streptavidin APC-Cy7 (1/500); cat. number 405208; validation: tested during reagent development

Identification of viable cells was done by fixable near-IR dead cell staining (Life Technologies).

Validation	Validation method is indicated in the table above. Some antibodies were validated by the manufacturer and information was extracted from their website.

Eukaryotic cell lines

Policy information about cell lines

Cell line source(s)	BHK-21 [C-13] (ATTCC CCL-10)
Authentication	The cell lines were not authenticated in our lab.
Mycoplasma contamination	Cell lines used for virus production were free of Mycoplasma contamination.
Commonly misidentified lines (See ICLAC register)	No commonly misidentified lines were used in this study.

Animals and other organisms

Policy information about studies involving animals; ARRIVE guidelines recommended for reporting animal research

Laboratory animals	P14 mice (Rag sufficient) expressing a transgenic T cell receptor specific for the glycoprotein 33-41 (gp33) epitope of the lymphocytic choriomeningitis (LCMV) virus presented on H-2Db 57 on a congenic CD45.1 background were bred and maintained at the ETH Phenomics Center. CD45.2 C57BL/6 were bred at the ETH Phenomics Center or purchased from Janvier Elevage (Saint Berthevin, France) or Charles River (United Kingdom). Six to sixteen-week-old male or female mice were considered young, mice >50 weeks of age were considered middle-aged and mice >70 weeks were considered aged. For all experiments where adoptive cell transfer was performed, aged mice were > 70 weeks old. All mice were bred and maintained under specific pathogen free (SPF) under SPF conditions at 24°C and 50% humidity, exposed to a 12:12h light/dark cycle and kept in individually ventilated cages (IVC) with autoclaved water and irradiated pellet feed provided ad libitum.
Wild animals	The study did not involve wild animals.
Field-collected samples	The study did not involve samples collected from the field.
Ethics oversight	Animal experiments were performed in accordance with institutional policies, Swiss and British federal regulations, and were

approved by the veterinary office of the Canton of Zürich (animal experimental permissions: 147/2014 and 115/2017) or by a local ethical review committee in UK (project license PPL 30/3388).

Note that full information on the approval of the study protocol must also be provided in the manuscript.

Flow Cytometry

Plots

Confirm that:

- [x] The axis labels state the marker and fluorochrome used (e.g. CD4-FITC).
- [x] The axis scales are clearly visible. Include numbers along axes only for bottom left plot of group (a 'group' is an analysis of identical markers).
- [x] All plots are contour plots with outliers or pseudocolor plots.
- [x] A numerical value for number of cells or percentage (with statistics) is provided.

Methodology

Sample preparation	Blood samples used for kinetics analysis were obtained from the tail vein. Spleens were obtained from PBS-perfused mice. Single cell splenocytes were prepared by meshing whole spleens through 70 µm strainers (BD Biosciences) using a syringe plunger. When cytokine production was assessed, CD8+ T cells within splenocytes were stimulated with 1 µg/ml of gp33 peptide in the presence of 10 µg/ml of Brefeldin A (Sigma-Aldrich) for 6 hours at 37°C. Identification of viable cells was done by fixable near-IR dead cell staining (Life Technologies). Erythrocytes were lysed by ACK lysis buffer treatment for 5 min at room temperature. Surface stainings were performed for 20 min at 4°C. For cytokine production analysis, cells were further fixed and permeabilized in 2× FACS Lysis Solution (BD Biosciences) with 0.08% Tween 20 (National Diagnostics, Chemie Brunschwig AG, Basel, Switzerland) for 10 min at room temperature, followed by intracellular staining for 30 min at room temperature in the dark. For the assessment of phosphoprotein expression from in vitro activated CD8+ T lymphocytes, surface staining was followed by fixation with pre-heated 4% paraformaldehyde (Sigma-Aldrich) for 10 min at 37°C, permeabilization with 90% of pre-cooled methanol for 30 min on ice and staining for 40 min at room temperature in the dark. All samples were washed and stored in PBS containing 2% FBS (Omnilab) and 5mM of EDTA (Sigma-Aldrich) before acquisition.
Instrument	Multi-parametric flow cytometric analysis was performed using Fortessa flow cytometer (BD Biosciences)
Software	Flow cytometry data were analyzed by Flowjo 10.7.1.
Cell population abundance	For bulk CD8 assays, CD8+ T cells were isolated from spleens of CD45.1 P14 mice using the EasySepTM Mouse CD8+ T cell Isolation Kit (StemCell, Grenoble, France) or the MojoSortTM Mouse CD8+ T cell Isolation Kit (BioLegend, Lucerna Chem AG, Luzern, Switzerland), following manufacturer's instructions. For analysis of specific cell subsets and CD8+ T cell enrichment, erythrocytes were lysed and remaining splenocytes were incubated with α-B220 biotin (RA3-6B2, BioLegend), and α-CD4 biotin (GK1.5, BioLegend) antibodies for 20 min at room temperature, followed by incubation with MojoSortTM streptavidin magnetic beads (BioLegend) for 5 min at room temperature, and further magnetic separation. After enrichment, cells were then stained for phenotypical markers, and subpopulations of interest were sorted on a FACS Aria cell sorter (BD Biosciences). Post-sort fractions were also acquired directly after sorting to check the purity. Flow cytometry analysis of adoptive transfer experiments was never limited by cell numbers of target cells.
Gating strategy	For all samples the following gating strategy was used: lymphocytes (SSC-A/FSC-A), exclusion of doublets (FSC-A/FSC-H), live cells (FSC-A/Live/Dead marker Near-IR). To identify congenitally marked P14 TCR transgenic CD8+ T cells, cells were gated for CD8 and for the congenic marker CD45.1.

- [x] Tick this box to confirm that a figure exemplifying the gating strategy is provided in the Supplementary Information.
